# Chronic Medical Conditions and Risk of Sepsis

**DOI:** 10.1371/journal.pone.0048307

**Published:** 2012-10-31

**Authors:** Henry E. Wang, Nathan I. Shapiro, Russell Griffin, Monika M. Safford, Suzanne Judd, George Howard

**Affiliations:** 1 Department of Emergency Medicine, University of Alabama School of Medicine, Birmingham, Alabama, United States of America; 2 Department of Emergency Medicine, Beth Israel Deaconess Medical Center, Boston, Massachusetts, United States of America; 3 Department of Epidemiology, School of Public Health, University of Alabama at Birmingham, Birmingham, Alabama, United States of America; 4 Division of Preventive Medicine, Department of Medicine, University of Alabama School of Medicine, Birmingham, Alabama, United States of America; 5 Department of Biostatistics, School of Public Health, University of Alabama at Birmingham, Birmingham, Alabama, United States of America; Oregon Health and Science University, United States of America

## Abstract

**Background:**

We sought to determine the associations between baseline chronic medical conditions and future risk of sepsis.

**Methods:**

Longitudinal cohort study using the 30,239 community-dwelling participants of the REGARDS cohort. We determined associations between baseline chronic medical conditions and incident sepsis episodes, defined as hospitalization for an infection with the presence of infection plus two or more systemic inflammatory response syndrome criteria.

**Results:**

Over the mean observation time of 4.6 years (February 5, 2003 through October 14, 2011), there were 975 incident cases of sepsis. Incident sepsis episodes were associated with older age (p<0.001), white race (HR 1.39; 95% CI: 1.22–1.59), lower education (p<0.001) and income (p<0.001), tobacco use (p<0.001), and alcohol use (p = 0.02). Incident sepsis episodes were associated with baseline chronic lung disease (adjusted HR 2.43; 95% CI: 2.05–2.86), peripheral artery disease (2.16; 1.58–2.95), chronic kidney disease (1.99; 1.73–2.29), myocardial infarction 1.79 (1.49–2.15), diabetes 1.78 (1.53–2.07), stroke 1.67 (1.34–2.07), deep vein thrombosis 1.63 (1.29–2.06), coronary artery disease 1.61 (1.38–1.87), hypertension 1.49 (1.29–1.74), atrial fibrillation 1.48 (1.21–1.81) and dyslipidemia 1.16 (1.01–1.34). Sepsis risk increased with the number of chronic medical conditions (p<0.001).

**Conclusions:**

Individuals with chronic medical conditions are at increased risk of future sepsis events.

## Introduction

Sepsis is the syndrome of microbial infection complicated by systemic inflammatory response, a process that may eventually lead to organ injury, shock and death. [1] Sepsis poses a significant burden upon the US healthcare system, resulting in an estimated 750,000 hospital admissions, 570,000 Emergency Department visits, 200,000 deaths and $16.7 billion in medical expenditures annually. [2], [3], [4] A prior study highlights the presence of regional variations in US sepsis mortality. [5].

Over the last century, the most significant public health gains in the United States have resulted from evidence-based risk stratification, detection and reduction efforts for common medical conditions such as cardiovascular disease and stroke. [6], [7], [8] Despite the national importance of the condition, progress at reducing the public health impact of sepsis has been relatively limited. A potential explanation is that current scientific and clinical initiatives tend to focus upon the acute care of sepsis after the onset of disease. Despite the presence of plausible pathophysiologic pathways as well as prevention and risk reduction strategies, few efforts have conceptualized sepsis as a predictable or preventable condition. [9], [10].

The first step in devising disease risk stratification or prevention strategies is to identify the characteristics of individuals at increased risk of developing the illness. A suitable design for characterizing the risk factors associated with sepsis is a population-based cohort with baseline information on each individual coupled with prospective longitudinal surveillance for incident sepsis events. [11] The Reasons for Geographic And Racial Differences in Stroke (REGARDS) study is one of the nation’s largest ongoing longitudinal cohort studies, encompassing 30,239 community-dwelling participants across the US. [12] The objective of this study was to describe the associations between baseline chronic medical conditions and future risk of sepsis in the REGARDS cohort.

## Methods

### Ethics Statement

This study was approved by the Institutional Review Board of the University of Alabama at Birmingham.

### Study Design

The study utilized a population-based longitudinal cohort design using the national REGARDS cohort.

### The REGARDS Cohort

The REGARDS study is one of the largest ongoing national cohorts of community-dwelling individuals in the US. [12] Designed to evaluate geographic and black-white stroke mortality variations, REGARDS includes 30,239 individuals ≥45 years old from across the United States. REGARDS encompasses representation from all regions of the continental US. Participant representation emphasizes the Southeastern US, with 20% of the cohort originating from the coastal plains of North Carolina, South Carolina and Georgia, and 30% originating from the remainder of North Carolina, South Carolina and Georgia plus Tennessee, Mississippi, Alabama, Louisiana and Arkansas. The cohort includes 41% African Americans, 45% men, and 69% individuals over 60 years old. The cohort does not include Hispanics.

REGARDS obtained baseline information on each participant from structured interviews and in-home visits. Baseline data for each participant include physical characteristics (height, weight), physiology (blood pressure, pulse, electrocardiogram), diet, family history, psychosocial factors and prior residences. The study also obtained biological specimens (blood, urine, etc.). On a semi-annual basis, the study contacts each participant to determine the date, location and attributed reason for all hospitalizations during the prior 6 months. If the participant has died, the study team interviewed proxies to ascertain the circumstances of the participant’s death. Follow-up on participants in this manner has occurred since 2003.

### Identification of Sepsis Events

We sought and reviewed all hospitalizations attributed by participants to a serious infection. Definitions for serious infections were based upon infection taxonomies developed by Angus, et al. [2] Two trained abstractors independently reviewed all relevant medical records to confirm 1) the presence of a serious infection on initial hospital presentation, and 2) the relevance of the serious infection as a major reason for hospitalization. The abstractors then identified clinical and laboratory information from the first 28-hours of hospitalization as well as outcomes for the hospitalization. The presence of a serious infection was based upon review of physician Emergency Department, hospital admission and hospital discharge records. We did not use laboratory, microbiological or radiographic information to define serious infections because these test results often have unclear connections with the overall clinical impression or course.

We used the international consensus definition of sepsis, consisting of presentation to the hospital with an infection plus two or more systemic inflammatory response syndrome (SIRS) criteria. [1] SIRS criteria included 1) heart rate >90 beats/minute, 2) fever (temperature >38.3°C or <36°C), 3) tachypnea (>20 breaths/min) or PCO_2_<32 mmHg, and 4) leukocytosis (WBC >12,000 or <4,000 cells/mm^3^ or >10% bands). We defined presentation to the hospital as the time of Emergency Department triage or admission to inpatient unit (for participants admitted directly to the hospital). To account for acute changes in the participant’s condition during early hospitalization, we used vital signs and laboratory test results for the initial 28-hours of hospitalization. The medical record review process did not include vital signs or laboratory findings at later time points, nor sepsis developing at later time period as a result of other major illness. For purposes of this analysis, we did not include organ dysfunction in the definition of sepsis.

All simple data discordances were resolved by consensus of abstractors with additional physician-level adjudication for more complex discordances. Initial review of 1,349 hospital records indicated excellent inter-rater agreement for the presence of a serious infection (kappa = 0.92) and the presence of sepsis (kappa = 0.90) upon hospital presentation.

### Definition of Covariates

Demographic characteristics evaluated in this analysis included age, sex, race, geographic region, income and education. Behavioral characteristics included tobacco and alcohol use. Tobacco use was defined as current, past and never. We defined alcohol use according to the National Institute on Alcohol Abuse and Alcoholism classification; i.e., moderate (1 drink per day for women or 2 drinks per day for men) and heavy alcohol use (>1 drink per day for women and >2 drinks per day for men). [13].

The parent REGARDS study was designed to oversample African-Americans and residents of the geographic regions referred to as the “Stroke Buckle” (coastal regions of North Carolina, South Carolina, and Georgia) and “Stroke Belt” (remainder of North Carolina, South Carolina, and Georgia; Alabama, Mississippi, Tennessee, Arkansas and Louisiana). We used the same geographic regions corresponding to “Stroke Buckle”, “Stroke Belt”, and “Non-Belt/Buckle.”

Evaluated chronic medical conditions included hypertension, diabetes, dyslipidemia, heart disease, atrial fibrillation, myocardial infarction, stroke, deep vein thrombosis, peripheral artery disease, chronic kidney disease and chronic lung disease. We defined hypertension as systolic blood pressure ≥140 mm Hg, diastolic blood pressure ≥90 mm Hg, or the use of antihypertensive agents. Diabetes included individuals with a fasting glucose ≥126 mg/dL, a non-fasting glucose ≥200 mg/dL, or the use of insulin or oral hypoglycemic agents. Dyslipidemia included individuals with self-reported high cholesterol or the use of lipid lowering medications.

Heart disease consisted of individuals with a self-reported history of myocardial infarction, coronary artery bypass grafting, or cardiac angioplasty or stenting, or baseline electrocardiographic evidence of myocardial infarction. We identified atrial fibrillation based upon participant self-report or baseline electrocardiographic evidence. Participants self-reported the prior history of stroke (including transient ischemic attacks), or deep vein thrombosis. Peripheral artery disease included a self-reported history of lower extremity arterial bypass or leg amputation.

Chronic kidney disease included those with an estimated glomerular filtration rate <60 ml/min/1.73 m2, calculated using baseline serum creatinine and the CKD-EPI equation. [14] REGARDS did not identify chronic lung diseases such as chronic obstructive pulmonary disease, emphysema, or asthma. Therefore we used self-reported pulmonary medications as a surrogate marker for chronic lung disease. Pulmonary medications included beta agonists, leukotriene inhibitors, inhaled corticosteroids, combination inhalers, and other pulmonary medications such as ipatropium, cromolyn, aminophylline and theophylline.

### Data Analysis

We compared demographic and clinical characteristics between sepsis and non-sepsis groups using the log-rank test for equality across strata. We used Cox proportional hazards regression to calculate hazard ratios and 95% confidence intervals for the association between each demographic and clinical factor and incident sepsis. For the Cox regression models, we defined person-time at risk as the time (days) from in-person examination to the first incidence of sepsis or the last follow-up interview, whichever came first. For the associations with each chronic medical condition, we adjusted the models for age, sex, race, education, geographic region, income, and smoking status. To assess the effect of comorbid burden upon sepsis risk, we created a Cox model evaluating the association between the number of chronic medical conditions and incident sepsis, adjusting for age, sex, race, geographic region, income, education and tobacco use. We confirmed the proportional hazards relationship for all regression models.

### Sensitivity Analysis

Due to the time lag in observations and medical record retrieval, we could not review medical records for 1,157 participants with a reported hospitalization for serious infection. Furthermore, these unexamined hospitalizations occurred across the observation period (2003–2011) and were not limited to select time periods. Therefore, in a sensitivity analysis we repeated the analysis excluding participants with reported hospitalizations for serious infection that had not yet been adjudicated.

## Results

Among the 30,239 REGARDS participants, from February 5, 2003 through October 14, 2011 we identified 2,157 hospitalizations for serious infection, encompassing 1,297 sepsis and 975 incident sepsis events. The most common infection types associated with incident sepsis cases were pneumonia, kidney and urinary tract infections, and abdominal infections. (Table 1) Pneumonia and other lung infections comprised over half of incident sepsis cases.

**Table 1:** Infection types associated with hospitalizations for sepsis.

Infection Type	Percentage of Incident Sepsis Hospitalizations (n = 975); n (%)
Pneumonia	427 (43.4)
Kidney and Urinary Tract Infections	155 (15.9)
Abdominal	133 (13.6)
Bronchitis, Influenza and other Lung Infections	84 (8.6)
Skin and Soft Tissue	71 (7.3)
Sepsis	63 (6.5)
Fever of Unknown Origin	14 (1.4)
Unknown/Other	14 (1.4)
Surgical Wound	6 (0.6)
Catheter (IV/Central/Dialysis)	5 (0.5)
Meningitis	3 (0.3)

The risk of incident sepsis was higher among older individuals. (Table 2) Whites were at higher risk of incident sepsis than blacks. Sepsis risk was also increased among those in the lowest education and income categories. While both current and past tobacco use were associated with increased incident sepsis risk, the risk was decreased with heavy or moderate alcohol use.

**Table 2:** Demographic and health behavioral characteristics between subjects who developed sepsis and those that did not.

	Sepsis (N = 975)	No Sepsis (N = 29,208)	Hazard Ratio (95% CI)	p-value*
**DEMOGRAPHICS**				
Age (%)				
45–54	4.9	11.9	Ref	<0.001
55–64	26.9	37.9	1.44 (1.04–2.00)	
65–74	36.2	31.9	2.29 (1.66–3.16)	
75+	32.0	18.2	3.87 (2.80–5.35)	
Gender (%)				
Male	52.5	44.6	1.30 (1.15–1.48)	0.12
Female	47.5	55.4	Ref	
Race (%)				
White	68.0	58.2	1.39 (1.22–1.59)	<0.001
Black	32.0	41.8	Ref	
Education (%)				
Less than high school	16.0	12.5	1.88 (1.54–2.29)	<0.001
High school graduate	28.7	25.8	1.52 (1.28–1.80)	
Some college	28.1	26.8	1.41 (1.19–1.67)	
College or higher	27.3	35.0	Ref	
Income (%)				
<$20k	24.7	17.9	1.78 (1.49–2.13)	<0.001
$20k–$34k	28.2	24.1	1.41 (1.19–1.67)	
$35k–$74k	25.9	29.7	Ref	
≥$75k	10.6	15.9	0.77 (0.61–0.96)	
Refused	10.6	12.4	1.07 (0.85–1.35)	
Geographic Region				
Stroke “Buckle”	21.6	20.9	1.23 (1.04–1.45)	0.04
Stroke “Belt”	37.8	34.5	1.22 (1.06–1.41)	
Non-[“Belt” or “Buckle”]	40.7	44.6	Ref	
**HEALTH BEHAVIORS**				
Tobacco Use (%)				
Current	17.8	14.5	1.85 (1.54–2.22)	<0.001
Past	48.7	39.9	1.64 (1.42–1.88)	
Never	33.5	45.6	Ref	
Alcohol Use (%)				
Heavy	4.0	4.0	0.89 (0.64–1.23)	0.02
Moderate	29.2	33.5	0.78 (0.67–0.89)	
None	66.8	62.5	Ref	

Total of 975 incident sepsis events among 30,239 participants in the REGARDS cohort.

* Estimated from a log-rank test.

**Figure 1:**
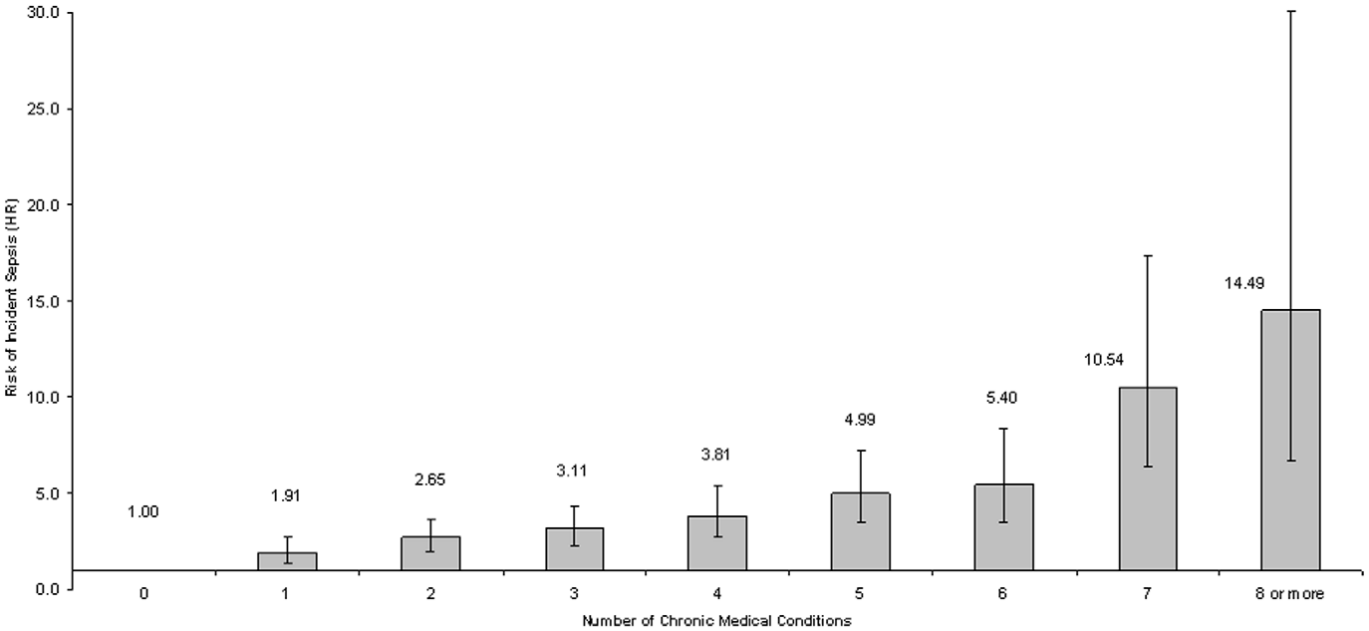
Adjusted risk of incident sepsis versus number of chronic medical conditions. Total of 975 incident sepsis events among 30,239 participants in the REGARDS cohort. Chronic medical conditions included hypertension, diabetes, dyslipidemia, coronary artery disease, atrial fibrillation, myocardial infarction, stroke, deep vein thrombosis, peripheral artery disease, chronic kidney disease and chronic lung disease. Hazard ratios adjusted for age, sex, race, education, income, geographic region, smoking status and alcohol use. P-value for test of trend <0.001.

All of the chronic medical conditions included in the analysis exhibited significant adjusted associations with incident sepsis. Chronic lung disease and chronic kidney disease exhibited the strongest adjusted associations with incident sepsis. (Table 3) The risk of incident sepsis was associated with the number of chronic medical conditions (p-trend <0.001). (Figure 1).

**Table 3:** Chronic medical conditions and associations with incident sepsis.

	Risk of Sepsis (per 1,000)			
Chronic Medical Condition	Among those with condition	Among those without condition	Unadjusted HR of Sepsis (95% CI)	Adjusted HR of Sepsis (95% CI)†
Chronic Lung Disease*	72.7	28.2	2.72 (2.33–3.17)	2.43 (2.05–2.86)
Peripheral Artery Disease	68.5	31.4	2.64 (1.96–3.55)	2.16 (1.58–2.95)
Chronic Kidney Disease	53.4	25.4	2.32 (2.04–2.64)	1.99 (1.73–2.29)
Myocardial Infarction	60.2	29.5	2.22 (1.87–2.64)	1.79 (1.49–2.15)
Diabetes	47.7	28.0	1.84 (1.61–2.11)	1.78 (1.53–2.07)
Stroke	56.5	30.5	2.08 (1.70–2.54)	1.67 (1.34–2.07)
Deep Vein Thrombosis	56.9	30.6	1.96 (1.58–2.43)	1.63 (1.29–2.06)
Coronary Artery Disease	52.7	27.7	2.05 (1.78–2.36)	1.61 (1.38–1.87)
Hypertension	37.6	24.3	1.61 (1.40–1.84)	1.49 (1.29–1.74)
Atrial Fibrillation	49.4	30.8	1.78 (1.47–2.14)	1.48 (1.21–1.81)
Dyslipidemia	38.2	29.3	1.30 (1.15–1.48)	1.16 (1.01–1.34)

Total of 975 incident sepsis events among 30,239 participants in the REGARDS cohort. Estimated from Cox proportional hazards regression and adjusted for gender, age, race, education, income, and smoking status.

† Adjusted for age, sex, race, education, income, geographic region, alcohol use, and smoking status.

* Chronic lung disease was defined as individuals reporting use of pulmonary medications.

In a sensitivity analysis, we repeated the analysis excluding 1,157 participants with reported serious infection hospitalizations that had not yet been reviewed or adjudicated, a figure expected to yield an additional 300 incident sepsis events; the associations were largely similar. Compared with incident sepsis individuals included in the analysis, the excluded individuals were older, more likely to be male, and had a higher number of chronic medical conditions. There were no racial differences between individuals included and excluded from the analysis.

## Discussion

This study confirms the association of baseline chronic medical conditions with the risk of future sepsis events. While prior studies have linked medical comorbidities with severity of sepsis or degree of organ dysfunction, there have been no efforts connecting these conditions at stable baseline with risk of future sepsis events. [11], [15], [16], [17], [18] The findings of this study may prove useful in sepsis care, pointing to risk detection, stratification and reduction as potential sepsis management strategies. Risk prevention and reduction strategies have proven effective for common medical conditions such as cardiovascular disease and stroke. [6].

We emphasize that this study identifies associations between baseline chronic medical conditions and sepsis but does not indicate a causal relationship. However, there are possible pathophysiologic connections between chronic medical conditions and the future risk of sepsis. Numerous common conditions have been associated with chronic inflammation, including obesity, diabetes, heart disease and smoking, among others. [19], [20], [21], [22], [23], [24] Inflammation plays a central role in sepsis pathophysiology, and chronic inflammation could raise the risk of progression to sepsis when subjected to a bacterial pathogen. [25] Chronic inflammation may also indicate individuals prone to developing a dysfunctional or exaggerated response to microbial infection. Associations between vascular disease and sepsis have not been previously described but are plausible given the role of endothelial dysfunction in sepsis pathophysiology. [26].

The notion of sepsis prevention is also plausible given the mutable nature of many of the risk factors identified in this study. For example, hypertension and dyslipidemia control are possible through pharmacotherapy and have resulted in large reductions in cardiovascular and cerebrovascular disease. [6] Glycemic control may limit sequelae of diabetes as well as the risk of pneumonia hospitalization. [27], [28] Smoking cessation is an important strategy for reducing cardiovascular risk and could yield similar benefits for sepsis risk reduction. [29] Outside of these conditions, aggressive vaccination strategies may provide another approach for curtailing disease. While seeming to overlap with risk factors already identified for other diseases, the identification of new relationships with sepsis is important because health behavior changes may be motivated differently by different medical conditions. Most importantly, our study confirms that an individual’s risk of sepsis is associated with the number of chronic medical conditions present. Therefore, if causal relationships were confirmed, mitigation of a combination of risk factors might reduce lifetime sepsis risk.

Our study offers additional perspectives of sepsis epidemiology and its risk factors. For example, half of the sepsis in this series involved infection types other than pneumonia. [30] Diabetes and chronic kidney disease have been associated with increased sepsis mortality; our study suggests that increased sepsis attack rates may partially explain the increased sepsis mortality in these subgroups. [31], [32] While recent studies highlight the increased risk of acute atrial fibrillation and stroke following a sepsis event, our study indicates that baseline atrial fibrillation and stroke are also precursors for sepsis. [33], [34] In contrast to studies suggesting a protective role from dyslipidemia, our study indicates that baseline dyslipidemia is clearly associated with an increased risk of sepsis. [35].

We also observed some unexpected findings. In contrast to prior studies, we did not detect a gender disparity in incident sepsis. [11] Although alcoholism has been implicated in immune system compromise, moderate alcohol use in this cohort appeared to be protective against sepsis, a finding consistent with studies suggesting similar protective effects against cardiovascular disease. [36], [37] Most importantly, in this study whites were at increased risk of sepsis compared with blacks, a finding that differs from published reports. [11], [38], [39] One potential explanation is that compared with whites, blacks in the REGARDS cohort may have underreported hospitalizations for serious infection. Also, prior studies of gender and sepsis were limited to hospitalized sepsis patients and did not evaluate the risk of sepsis among community dwelling individuals. We emphasize the preliminary nature of this observation and the need for more in-depth analysis to confirm this association and its possible explanations.

An important strength of our study was the use of a national population-based REGARDS cohort, which enabled us to connect baseline characteristics with the incidence of sepsis in individuals from across the US. Prior studies of sepsis epidemiology have been limited to single centers, patients admitted to intensive care units, or larger efforts based upon regional or national hospital discharge data. [2], [4], [40] Analyses based upon discharge data are limited by the accuracy of assigned diagnoses and have only limited ability to identify baseline patient characteristics or risk factors. In contrast, our study prospectively identified sepsis through the review of Emergency Department or hospital admission records, allowing for more certain identification of sepsis as a reason for (vs. sequelae of) hospitalization. While population-based sepsis studies have occurred in Denmark and other countries, in a prior study we identified two-fold regional variations in US sepsis mortality, underscoring the need for observations specific to the US. [5], [41].

### Limitations

Due to time lags in event reports and record retrieval, we could not review medical records for 1,157 individuals with reported serious infection hospitalizations, a figure expected to yield an additional 300 sepsis events. In the primary analysis we treated these observations as censored non-sepsis events. When repeating the analysis without these individuals, we identified largely similar results. Furthermore, compared with individuals included in the analysis, excluded subjects were older and exhibited a greater number of chronic medical conditions. Therefore, if we were to include these participants, we would likely observe even stronger associations with sepsis. The similar gender and race distribution between included and excluded cases provides assurance that medical record retrieval differences were not due to reporting or detection bias.

We did not examine severity variants of sepsis such as severe sepsis and septic shock because these conditions often develop later in the hospital course. Our study is relevant to the care of more advanced stages of sepsis because it identifies individuals at the earliest stages of disease, setting the stage for preventing subsequent severe sepsis and septic shock. We also did not study repeat sepsis events.

Participants reported hospitalizations for infection, potentially leading to under-identification of sepsis events due to recall or reporting biases. Our approach utilized prospective, systematic, dual review of hospital records using consensus definitions. While we were able to retrieve a large number of hospital records, the inability to retrieve select medical records may have biased the estimates.

Factors outside the scope of this analysis may potentially be associated with sepsis incidence; for example, biomarkers or genetic polymorphisms. Community level factors such as quality of care or antimicrobial resistance may also potentially influence sepsis risk. By design, the REGARDS cohort contains only African Americans and whites, and thus we could not examine associations with other racial groups or Hispanic ethnicity. While we examined income and education, other sociodemographic factors such as marital status may have altered sepsis risk. History of cancer was not ascertained by REGARDS. Our study indicates the presence of chronic medical conditions but not their quality of control. We could not detect potentially relevant comorbidities such as chronic liver disease. Because of the focus of this analysis on chronic medical conditions, we opted to limit examination of these and other confounders.

## Conclusions

Individuals with chronic medical conditions are at increased risk of developing future sepsis events.
